# 

**Published:** 1912-11-30

**Authors:** 


### LEICESTER ROYAL INFIRMARY.

The following workpeople's contributions (in round figures) have been received by the Leicester Royal Infirmary:—British United Shoe Machinery Co., £263; W. Tyler, Sons and Co., £208; Walker, Kempson and Stevens, Ltd., £146; Moore, Eady and Murcott Goode, Ltd., £130; Pool, Lorrimer and Tabberer, £122; Leavesby and North, £118; Gimson and Co., Ltd., £111.

Montreal.—At the annual meeting of the Foundling and Baby Hospital Lieutenant-Colonel Jeffrey H. Burland announced that he and his sisters would give £10,000 towards the purchase of a site and a new building, provided an equal amount was raised by others interested.

### Obituary.

LIEUTENANT-COLONEL WILLIAM OWEN, of the Indian Medical Service, has died at Weston-super-Mare. Educated at Trinity College, Dublin, he graduated in Arts in 1873 and in Medicine in 1889. He also became a Fellow of the Royal College of Surgeons of Ireland. His experience of actual service included the march from Kabul to Kandahar in 1880. He was eventually appointed in charge of the Government Opium Factory at Patna. His retirement took place in 1901.

MR. GEORGE WILLIAM ARMSTRONG, M.R.C.S., has died at Bournemouth at the age of seventy-two. The son of a London medical practitioner, Mr. Armstrong studied at King's College Hospital and qualified M.R.C.S. and L.S.A. in 1861. His chief work was done at Greenwich, where for some thirty years he held the post of resident medical officer and deputy superintendent at the Royal Hospital Schools, the medical charge of the officers and residents at the neighbouring Royal Naval College being also part of the duties of the appointment. It fell to his lot to act for a considerable time in place of his chief when on the sick-list, and his services were rewarded by a special pension from the Admiralty on his retirement, which took place about eleven years ago. This brief summary is enough to show in what high and wide esteem Mr. Armstrong was held in Greenwich society and among naval officers generally.

### Gifts and Bequests.

THE Chelsea Hospital for Women has received £157 10s. from Mr. F. G. Ivey towards its Rebuilding Fund.

THE Drapers' Company have sent £50 to the Trustees of the Lord Mayor Treloar Cripples' Hospital and College.

THE Chelsea Hospital for Women has received £105 from Mr. J. B. Hilditch and £100 from Sir Wm. Lancaster towards its Rebuilding Fund.

THE Chelsea Hospital for Women has received £100 from Mr. Thomas Joseph Whiffen and £52 10s. from Mr. Seth Taylor towards its Rebuilding Fund.

THE Chelsea Hospital for Women has received for its Rebuilding Fund £100 from Major-General R. C. B. Pemberton, C.B., C.S.I.; £50 from Major F. C. Foster and Mr. Miguel Lacroze.

THE Salters' Company have made their usual subscription of ten guineas to the After Care Association for poor persons discharged recovered from mental hospitals, Church House, Westminster.

THE Queen's Hospital for Children, Hackney Road, has received a further grant of £1,000 from the *Referee* Children's Fund towards the building and maintenance of the *Referee* Ward at the hospital's Little Folks Home at Bexhill.

MR. JOSEPH EDWARD HANNAH, of Totland Bay, I.W., retired civil engineer, who left estate of the gross value of £9,294, has, subject to two life interests, left the ultimate residue of his property to King Edward's Hospital Fund for London.

MISS ELIZABETH STRINGER, of St. Leonards, who left estate of the gross value of £262,361, has bequeathed £2,000 to the Liverpool Royal Infirmary, £1,000 each to the Convalescent Home for Women and Children, New Brighton, the Earlswood Asylum for Idiots, and the Hastings and St. Leonards Hospital.

MISS FRANCES ANN BASS, of Dalston Lane, N.E., who died on October 7, aged ninety-three, leaving estate of the gross value of £157,907, has left £500 each to the Earlswood Asylum for Idiots, the Hospital for Incurables, Putney, the City of London Hospital for Diseases of the Chest, the London Fever Hospital, the Royal Free Hospital, Gray's Inn Road, and the Metropolitan Hospital, Kingsland Road.

ALDERMAN ALFRED CORTIS, of Worthing, corn, seed, and coal merchant, first Mayor of Worthing, who left estate of the gross value of £131,840, has bequeathed £2,000 each to the Sussex County Hospital, Brighton, and the Worthing Hospital; £1,000 each to the Children's Care Society, Worthing, and to the Royal Blind Pension Society; and £500 to the Worthing District Nursing Association.

### BEQUESTS OF A SURGEON'S WIDOW.

MRS. A. M. JONES (widow of Professor Tom Jones, of Manchester, surgeon), who has left estate valued at £42,597, has left £500 to the University College of Wales, Aberystwyth, as an endowment for promoting the study of surgery; £1,000 to the Manchester Royal Infirmary, to provide a bed in the surgical ward; £300 to the Manchester Children's Hospital; £300 to the Christie Cancer Hospital (all of which last four legacies are to be associated with the name of her late husband); and £1,400 among various local philanthropic institutions. These legacies are subject to the estate's realising sufficient after payment of the personal legacies.

FLEET SURGEON FREDERICK WALTER STERICKER, R.N. (retired), M.D., M.R.C.S., L.S.A., of St. Helier, Jersey, has left estate valued at £19,856 gross, with net personalty £19,807.

### Appointments.

MR. H. W. HILLS has been appointed sixth assistant medical officer at Long Grove Mental Hospital.

MR. HERBERT W. PERKINS, F.R.C.S. (Eng.), D.P.H. (Lond.), has been appointed pathologist and bacteriologist to the Hampstead General and North-West London Hospital from January 1, 1913.

DR. JAMES PRIOR, a former house surgeon at Dewsbury Infirmary, has been appointed medical officer of the Staincliffe Workhouse, and Miss C. R. Lethem, resident medical officer at the Sheffield Children's Hospital, has been appointed lady resident assistant medical officer.

THE following re-appointments and appointments have been made at the Manchester Royal Infirmary: Re-appointments—Mr. W. H. Hey, F.R.C.S. (Eng.), assistant surgical officer, and Mr. John Gow, M.B., Ch.B. (Vict.), accident room house surgeon. Appointments—Dr. W. J. Reid, director of the cancer research laboratory; Mr. A. G. Wilkinson, M.B., Ch.B. (Vict.), Mr. T. H. Oliver, B.A. (Cantab.), M.B., Ch.B. (Vict.), and Mr. H. B. Willis, L.M.S.S.A. (Lond.), house physicians; Mr. N. Duggan, M.B., Ch.B. (Vict.), and Mr. N. Matthews, M.B., Ch.B. (Vict.), senior house surgeons; Mr. R. P. Stewart, M.B., Ch.B. (Vict.), M.R.C.S., L.R.C.P., and Mr. K. D. Bean, M.B., Ch.B. (Vict.), junior house surgeons; Mr. F. S. Bedale, M.A. (Cantab.), M.R.C.S., L.R.C.P., house surgeon to special departments.

THE REV. A. C. CRAWLEY-BOEVEY has been licensed to the chaplaincies of the General and Jaffray Hospitals, Birmingham, by the Bishop.

### Books Received.

J. B. LIPPINCOTT COMPANY: "Mouth Hygiene," by J. S. Marshall, M.D.

THE BROTHERHOOD PUBLISHING HOUSE: "Fifty Doctors against Alcohol."

STEPHEN SWIFT AND CO.: "The Doctor and His Work," by Chas. J. Whitby, M.D.

H. K. LEWIS: "Materia Medica and Pharmacy," by R. R. Bennett; "Massage and the Original Swedish Movements," by Kurre W. Ostrom; "Essays and Clinical Studies," by T. G. Crookshank, M.D. (Lond.).

P. S. KING AND CO.: "Hygiene for Health Visitors, School Nurses, and Social Workers"; "National Conference on the Prevention of Destitution."

T. FISHER UNWIN: "Hypnotism and Disease," by H. Crichton Miller, M.A., M.D.

LONGMANS AND CO.: "Manual of Surgical Treatment," Vol. II., by Cheyne and Burghard.

### AN ADVERTISING NOVELTY.

"THE Ballad of the 'Vaseline' (Household) Brigade" is the title of a ditty issued by the Chesebrough Manufacturing Company, proprietors of "Vaseline." The verses personify a set of the six "Vaseline" specialities as soldiers, giving each its rank and duty, General, Colonel, Major, Captain, Sergeant, Corporal. The whole is set to music, full folio size, and is handsomely produced. We understand that free copies are being sent to all who apply to the Company at 42 Holborn Viaduct, London, E.C.

